# The Cardiovascular Event Risk Associated with Tyrosine Kinase Inhibitors and the Lipid Profile in Patients with Chronic Myeloid Leukemia

**DOI:** 10.3390/hematolrep16010015

**Published:** 2024-03-12

**Authors:** María Nieves Saez Perdomo, Ruth Stuckey, Elena González-Pérez, Santiago Sánchez-Sosa, Paula Estupiñan-Cabrera, Sunil Lakhwani Lakhwani, José David González San Miguel, Nuria Hernanz Soler, Marina Gordillo, Gloria González Brito, María Tapia-Torres, Ana Ruano, Adrián Segura-Díaz, Hugo Luzardo, Cristina Bilbao-Sieyro, María Teresa Gómez-Casares

**Affiliations:** 1 Hospital Universitario de Gran Canaria Dr. Negrín, 35019 Las Palmas de Gran Canaria, Spain; mnieves.saez@dlongwood.com (M.N.S.P.); rstuckey@fciisc.es (R.S.); jsanchez@fciisc.es (S.S.-S.); paulaestcbr@gmail.com (P.E.-C.); aruaarr@gobiernodecanarias.org (A.R.); asegdia@gobiernodecanarias.org (A.S.-D.); hluzhen@gobiernodecanarias.org (H.L.); cbilsie@gobiernodecanarias.org (C.B.-S.); 2 Universidad de Las Palmas de Gran Canaria, 35001 Las Palmas de Gran Canaria, Spain; 3 Hospital Universitario de Canarias, 38320 La Laguna, Spain; slaklak@gobiernodecanarias.org (S.L.L.); ggonbri@gobiernodecanarias.org (G.G.B.); 4 Hospital Universitario Insular de Gran Canaria, 35016 Las Palmas de Gran Canaria, Spain; jdgonsan@gobiernodecanarias.org; 5 Hospital Universitario Nuestra Señora de Candelaria, 38010 Santa Cruz de Tenerife, Spain; nhersol@gobiernodecanarias.org; 6 Hospital José Molina Orosa, 35500 Las Palmas de Gran Canarias, Spain; mgormar@gobiernodecanarias.org; 7 Hospital General de La Palma, 38713 Santa Cruz de Tenerife, Spain; mtaptor@gobiernodecanarias.org; 8 Morphology Department, Universidad de Las Palmas de Gran Canaria, 35001 Las Palmas de Gran Canaria, Spain; 9 Department of Medical Sciences, Universidad de Las Palmas de Gran Canaria, 35001 Las Palmas de Gran Canaria, Spain

**Keywords:** chronic myeloid leukemia, cardiovascular, tyrosine kinase inhibitor (TKI), adverse event, lipid metabolism, imatinib, nilotinib

## Abstract

Background: Second- and third-generation tyrosine kinase inhibitors (TKIs) are now available to treat chronic-phase chronic myeloid leukemia (CP-CML) in the first and second line. However, vascular adverse events (VAEs) have been reported for patients with CML treated with some TKIs. Methods: We retrospectively evaluated the cumulative incidence (CI) and cardiovascular risk for 210 patients included in the Canarian Registry of CML. Result: With a mean follow up of 6 years, 19/210 (9.1%) patients developed VAEs, all of whom presented at least one cardiovascular risk factor at diagnosis. The mean time to VAE presentation was 54 months from the start of TKI treatment. We found a statistically significant difference between the CI for nilotinib-naïve vs. nilotinib-treated patients (*p* = 0.005), between dasatinib-naïve and dasatinib-treated patients (*p* = 0.039), and for patients who received three lines of treatment with first-line imatinib vs. first-line imatinib (*p* < 0.001). From the multivariable logistic regression analyses, the Framingham risk score (FRS) and patients with three lines of TKI with first-line imatinib were the only variables with statistically significant hazard ratios for VAE development. Significant increases in HDL-C and total cholesterol may also be predictive for VAE. Conclusions: In conclusion, it is important to estimate the cardiovascular risk at the diagnosis of CML as it can help determine whether a patient is likely to develop a VAE during TKI treatment.

## 1. Introduction

Chronic myeloid leukemia (CML) is a myeloproliferative neoplasm characterized by the expression of the BCR::ABL1 fusion protein, which exhibits constitutive tyrosine kinase activity [1]. In addition to defining the diagnosis, the detection of *BCR::ABL1* transcripts is essential for the molecular monitoring of patients with CML and assessing their response to tyrosine kinase inhibitor (TKI) treatment [2].

The introduction of second-generation TKIs (2G-TKIs)—nilotinib, dasatinib, and bosutinib—has significantly improved the treatment of chronic-phase CML (CP-CML). These 2G-TKIs have demonstrated superior response rates, leading to earlier cytogenetic and molecular responses compared to imatinib [3,4,5,6]. Moreover, when used as second-line therapy, 2G-TKIs can rescue a significant percentage of patients from imatinib treatment failure [7,8,9]. With the approval of the third-generation TKIs ponatinib, for patients in the chronic, accelerated, or blast phase who are resistant to dasatinib or nilotinib, and asciminib, for patients with CP-CML previously treated with two or more TKIs (with both also being approved for patients with the T315I kinase domain mutation), physicians now have a total of six TKIs available for the treatment of CP-CML.

During the follow up of patients with CML on TKI, it is important to periodically assess the appearance of treatment-related toxicities [10]. Each approved TKI has a unique off-target spectrum, meaning that physicians should consider patient comorbidities in order to choose the most appropriate treatment [11,12]. While different TKIs display diverse toxicities, some adverse events (AEs) are common, including facial and peripheral edema, diarrhea, skin rash, muscle cramps or myalgia, myelosuppressive effects, and glycometabolic alterations [13]. In the majority of cases, these AEs are not severe and can be reversed through dose interruptions or reductions [2,10].

Although imatinib has a well-documented and favorable safety profile, over the past decade, more serious toxicities have been reported in patients with CML treated with second- and third-generation TKIs, particularly vascular adverse events (VAEs) [14]. Subsequent clinical trials and retrospective studies have confirmed the risks of morbidity and mortality [3,6,15,16,17]. This is particularly important given that CML is typically diagnosed in older adults, with age as a CV risk factor for VAE [18,19]. Moreover, comorbidities such as arterial hypertension or diabetes mellitus are frequent among older patients. Thus, vascular safety is an important issue for patients with CML undergoing TKI treatment.

Reported VAEs associated with the use of second- and third-generation TKIs include severe occlusive arterial events, such as peripheral artery occlusive disease (PAOD), coronary artery disease, and ischemic cerebrovascular events. For instance, the 5-year follow-up study of ENESTnd indicated that among patients receiving nilotinib, 2.5% experienced PAOD, 3.9% experienced ischemic heart disease, and 1.4% experienced ischemic cerebrovascular events with the 300 mg dose, while 2.5%, 8.5%, and 3.2% experienced these events with the 400 mg dose. In comparison, the corresponding rates for patients receiving imatinib were 0%, 1.8%, and 0.4%, respectively [20]. In the initial results of the PACE clinical trial, cardiovascular, cerebrovascular, and peripheral vascular events that could possibly be related to treatment were observed in 2.2%, 0.7%, and 1.6% of patients receiving ponatinib, respectively [17]. In the DASISION trial, any-cause arterial ischemic events were reported in 5% of patients receiving dasatinib, compared to 2% of those receiving imatinib [6]. Similarly, in the BELA study, cardiac AEs were experienced by 4% of patients receiving bosutinib vs. 3% of patients receiving imatinib, with a prolonged QT interval being the most commonly reported VAE [21].

Outside of clinical trials, the actual incidence of VAEs in patients treated with TKIs is not extensively documented. In this retrospective real-life study, we aimed to assess the occurrence of VAEs in patients with CP-CML included in the Canarian CML Registry and examine the risk factors associated with VAE during TKI treatment.

## 2. Materials and Methods

### 2.1. Patient Cohort

The medical records of patients with CP-CML diagnosed and treated between 2009 and 2017 in the 7 hospitals of the Canary Islands, Spain, and included in the Canarian CML Registry (Registro Canario de Leucemia Mieloide Crónica, https://rclmc.es/rclmc/ (accessed on 1 December 2022)) were reviewed. Data were retrospectively collected on TKI treatment, concomitant medication, VAE occurrence, and cardiovascular risk factors (CVRFs), including age, gender, body mass index (BMI), smoking, arterial hypertension (AHT), and diabetes mellitus (DM).

Fasting lipid profiles in protein serum were collected from routine blood biochemistry analyses for total cholesterol (TC), low- and high-density lipoprotein cholesterol (LDL-C and HDL-C), and triglycerides (TG). Patients receiving lipid-lowering therapy (such as statins) at the TKI start were excluded from this study due to the possible effects on lipid profiles.

BMI was classified according to the World Health Organization classification as normal weight (BMI < 24.99), overweight (25 < BMI < 29.99), or obese (BMI ≥ 30) [22,23,24].

### 2.2. Statistical Analysis

The difference in the incidence of VAE between nilotinib-naïve patients (had received a TKI other than nilotinib) and patients who had received nilotinib as well as dasatinib-naïve patients (had received a TKI other than dasatinib) and patients who had received dasatinib for at least 98 months was estimated using Kaplan–Meier curves. The log-rank or Breslow test was applied to quantify differences in the time to the event (VAE) between groups.

To analyze the impact of the different TKIs according to the treatment line, (I) first-line imatinib (reference group) was compared against first-line nilotinib (II), first-line dasatinib (III), second-line nilotinib after first-line imatinib (IV), second-line dasatinib after first-line imatinib during at least 55 months (V), patients who received 2 lines of 2G-TKI (VI), second-line imatinib after a first-line 2G-TKI (VII), and patients who received 3 lines of treatment with imatinib as the first line during at least 30 months (VIII).

To estimate the 10-year cardiovascular risk, the Framingham risk score (FRS) was assessed at diagnosis and used to categorize patients into three groups: ≤10% likelihood of developing a VAE, 10–20% likelihood of developing a VAE, and ≥20% likelihood of developing a VAE [22].

A multivariable logistic regression analysis was applied to investigate the impact of covariates (risk factors, exposure to TKI, or previous VAE event) on the occurrence of the VAE as the response variable. The following risk factors at diagnosis were included in the model selection: FRS, cohort (I-VIII), and the presence of a previous VAE.

For comparison of the eight treatment groups, the differences in biochemical levels of lipids (total cholesterol, HDL-C, LDL-C, and triglycerides) between the time of diagnosis and after each treatment were analyzed using ANOVA with a Bonferroni post hoc test.

Statistical analyses were performed with IBM SPSS v.23 and the results were considered significant at *p* < 0.05.

## 3. Results

### 3.1. Patient Characteristics

Data were included from the Canarian CML Registry for 210 patients diagnosed with CP-CML and treated with a TKI. The median age at diagnosis was 52 years (range of 13–84 years) and 106 patients (50.5%) were male. In terms of CVRF, 92 patients (43.8%) received concomitant medication for AHT, 50 patients (23.8%) had DM (all of whom were receiving hypoglycemic medication), 19 patients (9.1%) were smokers, and 42 patients (20%) were ex-smokers. According to the BMI, 37 patients were classified as possessing a normal weight, 39 patients were overweight, and 55 were obese; 79 were unclassified due to insufficient information. According to the Framingham risk score (FRS), at the time of diagnosis, 147 patients (70%) were classified as possessing a low-risk FRS (<10% chance of suffering an atherosclerotic event), 38 patients (18.1%) as intermediate-risk (10–20% chance of suffering an atherosclerotic event), and 25 patients (11.9%) as high-risk (>20% chance of suffering an atherosclerotic event).

A total of 19 patients (9.1%) had a VAE while on TKI treatment, with the median age at the event of 70 years. Of these, 19 (100%) were known to have an underlying CVRF and 10 (52.6%) had received nilotinib as first- or second-line treatment (Table 1). The mean range of time to present a VAE from the start of the TKI treatment line was 53.9 months (range of 0.3–171 months).

### 3.2. Incidence Analysis

When the patients who presented VAE were distributed according to cohorts, the highest incidence of VAE was seen for the cohort of patients who received three lines of treatment with first-line imatinib (30%, cohort VIII, Table 2). The cumulative incidence for first-line dasatinib (cohort III) and first-line imatinib with second-line nilotinib (cohort IV), both at 12.5%, was higher than for first-line nilotinib (8.1%) or first-line imatinib (7.3%), although only one patient who received dasatinib in the first line had a VAE. No VAEs were registered in the cohorts of patients who received first-line imatinib with second-line dasatinib (cohort V), two 2G-TKI lines (cohort VI), or first-line 2G-TKI with second-line imatinib (cohort VII).

Analyzing the time to VAE according to first-line treatment with the Kaplan–Meier analysis, the VAE risk at 55 months was 3.8% for imatinib, 5.7% for nilotinib, and 25% for dasatinib (*p* = 0.06, log-rank; *p* = 0.025, Breslow).

**Figure 1:**
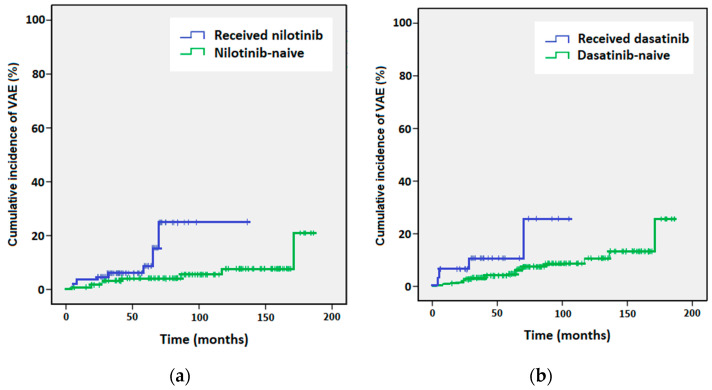
Kaplan–Meier curves of cumulative incidence of vascular adverse events (VAEs) for (**a**) patients who received nilotinib (blue line) versus nilotinib-naïve patients (green line), and (**b**) patients who received dasatinib (blue line) versus dasatinib-naïve patients (green line).

The Kaplan–Meier analysis of the time to VAE showed a statistically significant difference in the risk of VAE at 98 months between the nilotinib-naïve patients (had received a TKI other than nilotinib, 5.5%) and patients who had received nilotinib (25.8%, *p* = 0.029, Figure 1a). The risk of VAE at 98 months was also significantly different for the dasatinib-naïve patients (8.7%) vs. patients who had received dasatinib (25.6%, *p* = 0.039, Figure 1b).

Using an incidence pairwise comparison between the imatinib cohort (I) and the rest of the cohorts, only patients on second-line nilotinib after first-line imatinib (cohort IV) and patients with three lines of treatment with imatinib as the first line (cohort VIII) showed a statistically significant increase in the VAE risk (*p* = 0.029 and *p* < 0.0001, respectively, log-rank test). Comparison with the cohort of patients who received three lines of treatment with imatinib as the first line (VIII) was also statistically significant when the Breslow test was applied (*p* < 0.001).

### 3.3. Multivariable Logistic Regression

The evaluation of the impact of covariates (CVRF, exposure to TKI, or previous VAE) on the occurrence of VAEs as the response variable was carried out using multivariable logistic regression. The multivariable analysis showed that previous VAE was not significant for any of the cohorts. For the nilotinib-naïve/nilotinib and dasatinib-naïve/dasatinib cohorts, only FRS had a statistically significant association with VAE (Table 3). Considering the treatment cohorts, only the cohort of patients who received three lines of treatment with imatinib as the first line was significantly associated with occurrence of VAE (HR: 13.42, 95% CI: 2.15–83.78) when compared to first-line imatinib as a reference (cohort I).

Interestingly, FRS was associated with the occurrence of VAE for all the cohorts. Specifically, when comparing an FRS of 10–20 with ≤10, and ≥20 FRS with ≤10, the hazard ratios were 9.73 (95% CI: 2.43–38.99) and 7.40 (95% CI: 1.56–35.12), respectively.

### 3.4. Biochemical Lipid Analysis

A statistically significant elevation in TC and HDL-C and decrease in TG were observed for nilotinib-treated compared to nilotinib-naïve patients. However, only a statistically significant increase in HDL-C and a decrease in TG were observed for nilotinib-naïve patients before vs. after TKI treatment.

When comparing the lipid panels before and after treatment for the eight treatment cohorts, a statistically significant elevation in HDL-C (*p* < 0.05) and decline in TG (*p* < 0.01) were observed for first-line imatinib (cohort I), an elevation in HDL-C (*p* < 0.01) and a decrease in TG (*p* < 0.05) for first-line nilotinib (cohort II), an elevation in TC (*p* < 0.05) for first-line dasatinib (cohort III), an increase in TC (*p* < 0.01) and HDL-C (*p* < 0.01) for second-line nilotinib after first-line imatinib (cohort IV), an increase in LDL-C (*p* < 0.01) and decline in TG (*p* < 0.01) for second-line dasatinib after first-line imatinib (cohort V), an elevation in HDL-C (*p* < 0.01) for second-line imatinib after a first-line 2G-TKI (cohort VII), and a rise in TC (*p* < 0.05) for three lines of treatment with first-line imatinib (cohort VIII). In contrast, no significant differences in lipid levels were observed before and after treatment for two second-generation TKI treatment lines (cohort VI) (Table 4).

## 4. Discussion

In this retrospective study, the mean follow-up interval was 6 years (ranging from 3 months to 16 years). This extended follow-up period allowed late toxicities to be captured, such as the development of atherosclerotic lesions. This was one advantage of our study, since prior reports of VAE for patients with CML on TKI have predominantly been based on clinical trials with follow-up periods of less than 5 years. For example, one patient developed a VAE after 14.3 years of first-line imatinib treatment. However, it is important to acknowledge that the sample size for some of the treatment cohorts included in the analyses was relatively small. This limitation reduces the statistical power of this study.

The incidence of VAEs in patients with CP-CML is as yet unknown and it remains unclear if the incidence differs from that of the age-matched general population. Indeed, in our study, we observed a similar incidence of VAEs in our cohort of 210 patients with CP-CML who were treated with TKIs (9.1%) compared to the age-matched general population (10–20% for adults aged 50–70 years [25]), even though the patients in our cohort had CML and many had one or more CVRFs.

Nevertheless, consistent with prior reports, our analysis confirmed a higher incidence of VAEs in patients who were treated with nilotinib compared to those receiving other TKIs [15,16,18,26]. Specifically, a higher incidence of VAEs was observed for second-line nilotinib after first-line imatinib vs. first-line nilotinib (12.5% vs. 8.1%, respectively), and statistically significant differences in VAE were also observed when comparing nilotinib-naïve versus nilotinib-treated patients, and patients who received three lines of treatment with first-line imatinib versus first-line imatinib. When compared with patients on first-line imatinib, a statistically significant difference was only observed for patients who received nilotinib as second- or third-line TKI treatment, as previously reported [20]. Moreover, the multivariable logistic regression analysis did not identify treatment with nilotinib as a significant risk factor for developing a VAE. These results may suggest a latency period of nilotinib treatment before VAEs develop [14]. Indeed, the mean time to VAE from the TKI start was 54 months (for the whole cohort).

The results of the multivariable logistic regression analysis revealed that only one variable, the FRS, obtained a statistically significant hazard ratio for the development of a VAE. Interestingly, the hazard ratio for the 10–20 FRS group was higher than the hazard ratio for the ≥20 FRS group when compared to the ≤10 FRS group. This difference may be due to the closer follow up of patients with a higher FRS and/or use of prophylaxis (such as aspirin or statins). This hypothesis might also explain why previous VAE was not found to be a risk factor for any of the treatment cohorts. However, no significant difference in VAE incidence was observed between the 10–20 FRS and ≥20 FRS groups regardless of the treatment cohort (data not shown). Similarly, other studies have shown that the FRS or other CV risk stratification tools, such as the HFA/ICOS or the Systematic Coronary Risk Evaluation (SCORE), may be useful for predicting the risk of VAE for patients with CML on TKI treatment [27,28,29]. It is noteworthy that all patients with VAEs presented at least one CVRF at diagnosis. This observation underscores the importance of considering CV risk factors when choosing an appropriate TKI.

Consistent with previous studies [20,30,31], changes to lipid metabolism were observed with the TKI treatment start. Indeed, the first study of TKI effects on lipid profiles reported that of six patients with CML and hypercholesteremia (two of whom also had hypertriglyceridemia), all six had a long-lasting normalization of lipid levels while receiving imatinib [31]. For patients who received first-line imatinib in our study (cohort I), a decline in TG was also observed, although HDL-C levels increased. Meanwhile, a significant rise in TC and HDL-C and a decrease in TG were observed for patients who received nilotinib; contrary to previous reports, modification in LDL-C levels was not observed [30]. A significant increase in LDL-C levels was only identified for patients who received first-line imatinib with second-line dasatinib (cohort V), although only three patients received this treatment combination. Nevertheless, it remains to be determined if these changes in lipid metabolism were responsible for the development of a VAE.

Taken together, our observations are consistent with prior reports [18,20,32,33] that suggest patients at risk of developing VAEs during TKI therapy might be identifiable at the time of diagnosis. Our results support the estimation of the cardiovascular risk at the time of diagnosis in order to choose the most appropriate first-line treatment to prevent the appearance of VAEs [32]. For patients with intermediate and high cardiovascular risk, physicians should establish a prevention plan to diminish the cardiovascular risks throughout the TKI treatment [34]. The plan may include anti-ischemic/hypertensive drugs, statins, optimum control of DM (as needed), and increased patient monitoring, including perhaps lipid profiles. However, it is also important to monitor patients with low cardiovascular risk during TKI treatment to re-adapt strategies when required (i.e., if the cardiovascular risk increases to intermediate or high).

Finally, a potential strategy to reduce VAE risk among patients with CML could be the discontinuation of TKI treatment [35]. The global time of TKI treatment is an important variable when considering if a patient is eligible for TKI discontinuation, as is the duration of a deep molecular response, both associated with a higher probability of treatment-free remission [36].

## 5. Conclusions

A higher incidence of VAEs was observed for patients treated with nilotinib compared to patients treated with other TKIs and for patients who received three lines of TKI treatment.

We consider that the estimation of the cardiovascular risk at diagnosis is a powerful tool to determine if a patient with CML is likely to develop a VAE during TKI treatment. To this end, our results reveal that the FRS is useful. Changes in HDL-C and total cholesterol levels with TKI treatment may also be predictive for VAE. The assessment of cardiovascular risk at the time of diagnosis would allow hematologists and other specialists, as part of a multidisciplinary team, to develop a prevention plan to minimize the incidence and severity of VAEs.

## Figures and Tables

**Table 1:** VAE patient cases.

No.	Gender	Age (Years)	Adverse Event	Treatment Group	Months on TKI to Onset	Dose at Onset	Received NIL	CVRF at Baseline
1	F	61	PAOD with ischemic ulceration	First-line IMA (I)	26	IMA at 400 mg/24 h	No	DM, AHT
2	M	86	Intestinal occlusion with ischemic origin	First-line IMA (I)	18	IMA at 200 mg/24 h	No	Smoker, AHT
3	F	70	CVA	First-line IMA (I)	136	IMA at 400 mg/24 h	No	DM, AHT, hyperlipidemia
4	M	64	Acute myocardial infarction	First-line IMA (I)	42	IMA at 400 mg/24 h	No	DM, hyperlipidemia, smoker, ischemic cardiomyopathy
5	F	68	CVA	First-line IMA (I)	87	IMA at 400 mg/24 h	No	Smoker, AHT, hyperlipidemia
6	M	86	Lower limb amputation	First-line IMA (I)	117	IMA at 400 mg/24 h	No	Smoker, hyperlipidemia, acute myocardial infarction
7	M	83	PAOD	First-line IMA (I)	171	IMA at 100 mg/24 h	No	AHT
8	M	77	Coronary artery ischemic cardiomyopathy	First-line IMA (I)	3	IMA at 400 mg/24 h	No	DM, ex-smoker, AHT, hyperlipidemia
9	F	74	AION	First-line NIL (II)	31	NIL at 600 mg/24 h	Yes	DM, AHT
10	F	67	Unstable angina	First-line NIL (II)	65	NIL at 300 mg/24 h	Yes	DM, AHT
11	M	65	Intermittent claudication	First-line NIL (II)	23	NIL at 400 mg/24 h	Yes	Smoker, AHT, CVA
12	M	72	CVA	First-line DAS (III)	28	DAS at 100 mg/24 h	No	DM, ex-smoker, AHT
13	F	80	CVA	First-line IMA and second-line NIL (IV)	65	NIL at 600 mg/24 h	Yes	DM, ex-smoker, AHT, acute myocardial infarction
14	F	86	Toe amputation	First-line IMA and second-line NIL (IV)	58	NIL at 800 mg/24 h	Yes	DM, AHT
15	M	59	Acute myocardial infarction	First-line IMA and second-line NIL (IV)	8	NIL at 600 mg/24 h	Yes	DM, AHT, smoker, hyperlipidemia
16	F	56	Intermittent claudication	First-line IMA and second-line NIL (IV)	70	NIL at 600 mg/24 h	Yes	DM, AHT
17	F	62	CVA	Three treatment lines with first-line IMA (VIII)	70	DAS at 50 mg/24 h	Yes	DM, AHT
18	F	73	Lower limb amputation	Three treatment lines with first-line IMA (VIII)	5	NIL at 600 mg/24 h	Yes	DM, AHT
19	M	50	AION	Three treatment lines with first-line IMA (VIII)	0.3	IMA at 400 mg/24 h	Yes	Ex-smoker, AHT

AHT, arterial hypertension; AION, anterior ischemic optic neuropathy; CVA, cerebrovascular accident; CVRF, cardiovascular risk factor; DAS, dasatinib; DM, diabetes mellitus; F, female; IMA, imatinib; M, male; NIL, nilotinib; No., patient number; PAOD, peripheral arterial occlusive disease; TKI, tyrosine kinase inhibitor.

**Table 2:** Distribution of patients who developed a vascular adverse event (VAE) across the cohorts.

Cohorts	Patients with VAE	Patients without VAE	Total	%
Nilotinib-naïve	9	117	126	7.1
Patients who received nilotinib	10	74	84	11.9
Total	19	191	210	
Dasatinib-naïve	16	165	181	8.8
Patients who received dasatinib	3	26	29	10.3
Total	19	191	210	
First-line imatinib (I)	8	101	109	7.3
First-line nilotinib (II)	3	34	37	8.1
First-line dasatinib (III)	1	7	8	12.5
First-line imatinib with second-line nilotinib (IV)	4	28	32	12.5
First-line imatinib with second-line dasatinib (V)	0	8	8	0
Two 2G-TKI lines (VI)	0	3	3	0
First-line 2G-TKI with second-line imatinib (VII)	0	3	3	0
Three lines of treatment with first-line imatinib (VIII)	3	7	10	30.0
Total	19	191	210	

2G-TKI, second-generation tyrosine kinase inhibitor.

**Table 3:** Multivariable logistic regression.

Treatment Group	Risk Factor	Effect	HR	95% CI
Nilotinib-naïve	FRS	10–20 vs. ≤10	7.75	2.20–26.37
*vs.* nilotinib	FRS	≥20 vs. ≤10	5.75	1.38–23.98
	Prior VAE	Yes vs. no	2.71	0.77–9.48
	Cohort	Nilotinib vs. nilotinib-naïve	2.34	0.83–5.57
Dasatinib-naïve	FRS	10–20 vs. ≤10	6.87	1.99–23.61
*vs.* dasatinib	FRS	≥20 vs. ≤10	5.70	1.33–24.50
	Prior VAE	Yes vs. no	3.04	0.86–10.70
	Cohort	Dasatinib vs. dasatinib-naïve	2.49	0.67–9.23

CI, confidence interval; FRS, Framingham risk score group; VAE, vascular adverse event.

**Table 4:** Biochemical risk factors for VAEs in patients under tyrosine kinase inhibitor treatment.

					Cohort			
mg/dL	I	II	III	IV	V	VI	VII	VIII
TC Dx (range)	175 (63–326)	190 (99–265)	151 * (85–243)	168 ** (88–278)	159 (132–207)	141 (109–158)	198 (142–274)	156 * (92–227)
TC (range)	167 (76–288)	195 (126–286)	162 * (118–203)	188 ** (91–264)	219 (182–248)	172 (164–188)	202 (131–285)	193 * (164–254)
HDL Dx (range)	40 * (21–75)	42 ** (21–66)	-	45 ** (17–102)	44 (23–171)	-	34 ** (31–37)	38 (25–57)
HDL (range)	45 * (18–83)	54 ** (35–130)	-	56 ** (34–93)	52 (35–64)	-	67 ** (41–78)	52 (35–74)
TG Dx (range)	179 ** (12–814)	161 * (41–689)	135 (97–218)	160 (37–510)	427 ** (83–1024)	120 (75–124)	142 (114–170)	145 (90–268)
TG (range)	132 ** (29–367)	109 * (45–286)	103 (68–254)	126 (31–471)	187 ** (89–338)	124 (94–147)	145 (65–225)	121 (56–166)
LDL Dx ((range)	101 (9–237)	105 (43–165)	-	102 (22–196)	67 ** (46–114)	-	96 (78–113)	98 (49–136)
LDL (range)	90 (20–227)	116 (49–184)	-	116 (22–181)	134 ** (107–149)	-	79 (161–199)	117 (97–167)

Dx, diagnosis; HDL, high-density lipoprotein; LDL, low-density lipoprotein; TC, total cholesterol; TG, triglycerides. * *p* < 0.05, ** *p* < 0.01. HDL and LDL data were not available for cohorts III and VI.

## Data Availability

The data that support the findings of this study are available from the corresponding author upon reasonable request.
